# Gamma oscillations provide insights into cortical circuit development

**DOI:** 10.1007/s00424-023-02801-3

**Published:** 2023-03-03

**Authors:** Sebastian H. Bitzenhofer

**Affiliations:** Institute of Developmental Neurophysiology, Center for Molecular Neurobiology, Hamburg Center of Neuroscience, University Medical Center Hamburg-Eppendorf, Falkenried 94, 20251 Hamburg, Germany

**Keywords:** Gamma oscillations, Prefrontal cortex, Development, Inhibition, Neuropsychiatric disorders

## Abstract

Rhythmic coordination in gamma oscillations provides temporal structure to neuronal activity. Gamma oscillations are commonly observed in the mammalian cerebral cortex, are altered early on in several neuropsychiatric disorders, and provide insights into the development of underlying cortical networks. However, a lack of knowledge on the developmental trajectory of gamma oscillations prevented the combination of findings from the immature and the adult brain. This review is intended to provide an overview on the development of cortical gamma oscillations, the maturation of the underlying network, and the implications for cortical function and dysfunction. The majority of information is drawn from work in rodents with particular emphasis on the prefrontal cortex, the developmental trajectory of gamma oscillations, and potential implications for neuropsychiatric disorders. Current evidence supports the idea that fast oscillations during development are indeed an immature form of adult gamma oscillations and can help us understand the pathology of neuropsychiatric disorders.

## Introduction

The temporal coordination of action potentials fired by a group of neurons is important for activity in neuronal networks. Particularly in networks with weak excitatory synapses, such as the mammalian cerebral cortex, synchronization of neurons is critical. Activation of an individual excitatory synapse rarely suffices to trigger an action potential in a cortical neuron and only the summation of several inputs close in time can push the membrane potential above the action potential threshold [51]. Non-linear effects of synaptic integration in neuronal dendrites further enhance the impact of synchronous inputs [51]. Therefore, the impact of neuronal activity strongly depends on its temporal coordination within the local network.

Temporal organization of neuronal activity in oscillatory rhythms is a commonly observed phenomenon in the cerebral cortex [15]. Oscillatory rhythms can be detected in recordings of individual neurons, but more commonly they are investigated with extracellular recordings of field potentials, obtained by methods such as electroencephalography, electrocorticography or intracranial electrophysiology. These methods provide information about the activity of a neuronal network without the need to record from each individual neuron. The features of oscillatory rhythms provide information about the underlying network.

Considerable attention has been given to gamma oscillations, rhythmic activity with a frequency in the range of 30–100 Hz. Cortical gamma oscillations typically occur when an area is activated and they are generated by the interaction of excitatory neurons and specific populations of inhibitory interneurons [16].

Altered gamma oscillations in the adult cortex have been associated with several neuropsychiatric disorders and have been suggested as a diagnostic biomarker [43, 50, 53]. Many of these disorders have a developmental origin [31, 34, 44]. This raises the questions whether gamma oscillations are already impaired during development and whether they can be linked to the progression of these disorders. Addressing these questions requires a general understanding of the physiological development of gamma oscillations.

Oscillations in the low gamma frequency range have been reported for several cortical areas during postnatal development in rodents [3, 14, 37, 59]. Further, immature gamma oscillations are altered in mouse models of neuropsychiatric disorders already during early postnatal development [21, 23]. However, some properties of these immature gamma oscillations are different from their adult counterpart: they appear in brief bursts of 100–300 ms, have a lower amplitude and a lower frequency compared to adult gamma oscillations in the cortex [59]. This raises the question whether fast oscillations during development represent an immature form of adult gamma oscillations or whether they are mechanistically different.

This review aims to summarize recent progress regarding the questions raised above with a focus on the development of gamma oscillations in the prefrontal cortex of mice. The review starts with a brief summary of the current knowledge on gamma oscillations in the adult cortex, followed by what is known about their development and their impairment in neuropsychiatric disorders. At the end, the role of gamma oscillations for the activity-dependent refinement of cortical networks is discussed.

## Gamma oscillations in the adult cerebral cortex

**Fig. 1:**
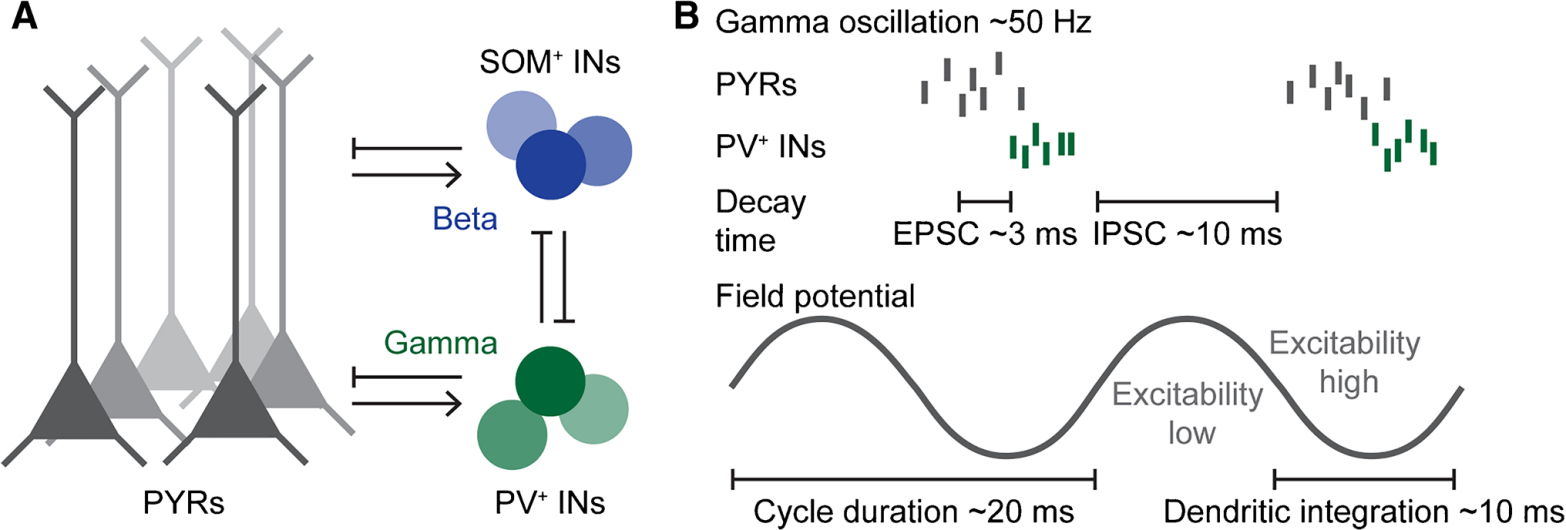
Gamma oscillations in the cerebral cortex. (**A**) Fast oscillatory rhythms are generated by the interaction of excitatory pyramidal neurons and inhibitory interneurons. Gamma oscillations at ~ 60 Hz are associated with PV^+^ interneurons, where slower beta oscillations are associated with SOM^+^ interneurons. (**B**) The rhythmic interactions of pyramidal neurons and PV^+^ interneurons result in alternating windows of high and low excitability. The cycle duration is largely defined by the duration of inhibitory postsynaptic potentials which define the duration of a break in the firing of pyramidal neurons. PYRs — pyramidal neurons, PV^+^ INs — parvalbumin-expressing interneurons, SOM^+^ INs — somatostatin-expressing interneurons, EPSC — excitatory postsynaptic potential, IPSC — inhibitory postsynaptic potential

The precise frequency of gamma oscillations depends on the network of interest but typically is in the range of 30–100 Hz in the cerebral cortex. This corresponds to a cycle duration of 10 to 33 ms. Several excellent reviews discuss the mechanisms and the role of gamma oscillations in the adult cortex [16, 27, 52]. Briefly, typical cortical gamma oscillations occur at a frequency of ~ 60 Hz and are generated by interactions of excitatory pyramidal neurons and inhibitory fast-spiking interneurons that express parvalbumin (PV^+^) (Fig. 1A) [6, 18]. Oscillatory activity in beta/low gamma frequency depends on interactions between pyramidal neurons and regular-firing somatostatin-expressing interneurons (SOM^+^) [20]. Gamma oscillations evoked by gratings in the visual cortex have a peak frequency at ~ 40 Hz and depend on SOM^+^ but not PV^+^ interneurons indicating that the specific features of the activated microcircuit in each area can lead to variations in oscillatory frequency [1, 55].

The activity of specific neuronal populations is concentrated at specific phases of a gamma cycle (Fig. 1B) [27]. When a sufficiently large group of pyramidal neurons fires together, they activate nearby PV^+^ interneurons which provide inhibitory feedback to the local network. PV^+^ interneurons target the cell bodies of pyramidal neurons and are therefore ideally positioned to block the generation of action potentials. The high connectivity of PV^+^ interneurons to nearby pyramidal neurons results in a break of firing that lasts until inhibition fades off, pyramidal neurons can fire again, and the cycle repeats. A key parameter defining the duration of this break and thereby the duration of a gamma cycle is the time constant of inhibitory synaptic potentials. This is largely determined by the decay time constant of GABA-A receptors, which is in the range of 10 ms in the adult cortex [32].

The temporal organization of neuronal firing to specific phases of each gamma cycle concentrates activity of the local excitatory population to a time window of about 10 ms [27]. This synchronization allows for the temporal integration of excitatory synaptic inputs, mainly mediated by AMPA receptors, with a decay time constant of about 3 ms [51]. Pyramidal neurons optimally integrate inputs to their distal dendrites that are within ~ 10 ms, whereas inputs closer to the soma are optimally integrated for a time window of ~ 50 ms [56]. The dendrites of pyramidal neurons preferentially receive lateral and top-down inputs, indicating that gamma oscillations could provide the optimal temporal structure to integrate sensory information with contextual information [48, 54]. This indicates that pyramidal neurons might be particularly receptive to dendritic input during gamma oscillations. Thus, synchronization of neuronal activity in gamma frequency modulates the processing in cortical networks.

## Development of cortical gamma oscillations

The molecular differentiation of diverse neuronal subtypes and their arrangement into cortical layers during embryonic development is mostly guided by molecular factors governed by genetic programs [12]. Excitatory neurons originate from the subventricular zone and undergo radial migration to form the cortex in an inside-out progression, whereas interneurons originate from the ganglionic eminence and migrate tangentially into the cortex [30, 41]. Once all cortical neurons have reached their final destination around birth in rodents, a crucial step in cortical development is completed. But another crucial phenomenon is just about to start: neurons become electrically active, expand their dendrites and axons, and form chemical synaptic connections [34, 39]. This process transforms an accumulation of segregated neurons into a network capable of coordinating its activity.

Initially, cortical neurons possess low spontaneous activity and fire relatively slow action potentials which rapidly mature during the first postnatal weeks [39, 49]. Weak synaptic connections during early development result in weak coordination of neuronal activity, but the gradual increase of synapse density and strength during the first postnatal weeks leads to a steep rise of coordinated activity patterns in cortical networks [4, 5, 34, 39, 42]. Initially, temporally coordinated activity is short-lived, with brief bursts interrupting periods of electrical silence. These early activity patterns, so-called spindle bursts, emerge towards the end of the first postnatal week, have been described in several cortical areas, last for 2–3 s, and organize activity in oscillations with frequencies from 4 to 12 Hz [14, 42, 59]. Spindle bursts organize neuronal activity locally, but also coordinate activity between areas [2, 14].

**Fig. 2:**
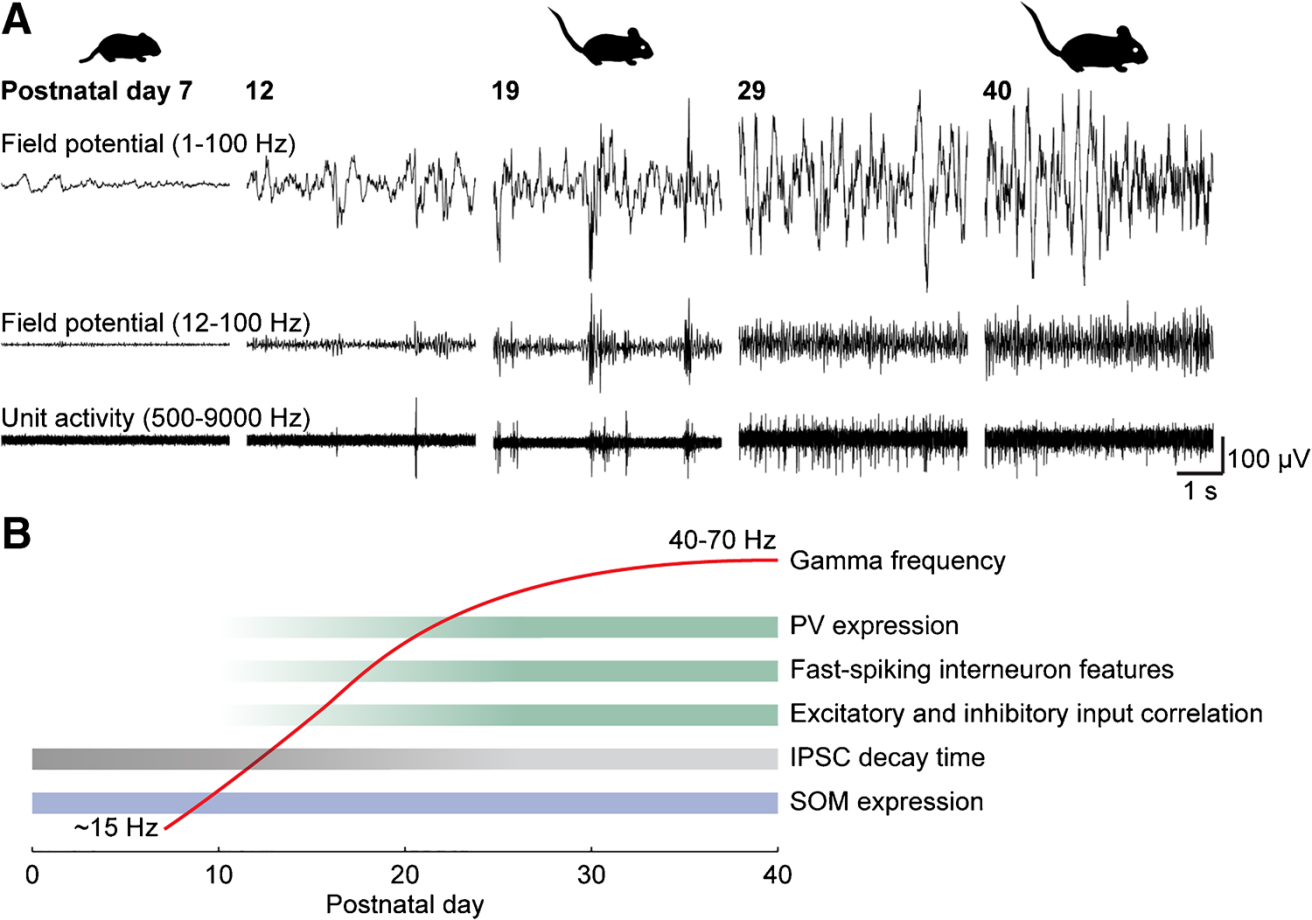
Gamma oscillations increase in frequency during postnatal development. (**A**) Recordings of the local field potential and unit activity in the prefrontal cortex of mice from postnatal day 7 to 40. The plot was made using data from a previous publication [10]. (**B**) Schema illustrating how the developmental increase in the frequency of gamma oscillations correlates with the maturation of inhibition, particularly of fast-spiking PV^+^ interneurons. PV — parvalbumin, SOM — somatostatin, IPSC — inhibitory postsynaptic potential

However, it is only at the beginning of the second postnatal week that oscillations at faster frequencies start to emerge (Fig. 2A) [10, 14, 37]. Initially, short-lived rhythmic synchronization of neuronal activity at the low end of the gamma frequency range occurs nested into spindle bursts [14]. Simultaneous recordings of synaptic inputs to single neurons and local field potentials in the prefrontal cortex revealed that particularly excitatory inputs to interneurons strongly correlate with these developmental gamma oscillations [11]. Notably, in primary sensory areas of the cortex, there is a distinct pattern of high-frequency oscillations that occurs transiently during the first postnatal week, i.e. early gamma oscillations, that are driven by excitatory input from the thalamus and are mechanistically different from adult gamma oscillations [46].

Layer-specific optogenetic activation identified pyramidal neurons in layer 2/3, but not layer 5/6, as the main driver of developmental gamma oscillations in the prefrontal cortex of mice [8]. The developmental trajectory of gamma oscillations triggered by such optogenetic stimulation is consistent with spontaneous gamma oscillations [10]. They emerge in the second postnatal week with initially low amplitude and frequency at about 15 Hz, which would be considered beta frequency according to the frequency bands defined for adults [8, 10, 14]. Amplitude and frequency consistently increase during development until they plateau at a level typical for adult gamma oscillations around postnatal day 25 (Fig. 2B) [10]. This increase in gamma frequency correlates well with the maturation of PV^+^ fast-spiking interneurons [10]. It also matches the development of balanced ratios of excitatory and inhibitory inputs onto pyramidal neurons, which largely depends on PV^+^ interneurons and might be required for the frequency modulation of gamma oscillations [6, 25, 58]. The frequency of gamma oscillations in the visual cortex shows a similar increase during postnatal development [19, 35].

Despite the slower frequency, similarities in the mechanism and the monotonic increase of frequency with age indicates that fast oscillatory activity during development represents an immature form of adult gamma oscillations. The exact mechanisms that lead to the increase in frequency are not entirely clear, but a dependency on the maturation of fast-spiking PV^+^ interneurons and the acceleration of the kinetics of inhibitory postsynaptic potentials can be postulated [10, 39]. In the adult cortex, SOM^+^ and PV^+^ interneurons are involved in the generation of slow and fast gamma oscillations [20]. While SOM^+^ interneurons are relatively stable in numbers, PV^+^ interneurons increase the expression of their typical marker PV and start to develop their fast-spiking characteristics during postnatal development [10]. Therefore, a developmental shift of the relative contribution to inhibitory feedback from SOM^+^ to PV^+^ interneurons might also contribute to the increase in gamma frequency.

## Developing gamma oscillations and neuropsychiatric disorders

Impaired gamma oscillations in the cortex are associated with a range of neuropsychiatric disorders in adults [43, 50, 53]. Dysfunctions of PV^+^ interneurons and failed integration into cortical microcircuits underlie altered gamma oscillations [24, 29, 50]. Deficient gamma oscillations in the prefrontal cortex have been linked to cognitive symptoms which are common in neuropsychiatric disorders and their rescue can alleviate cognitive impairment in mouse models [24, 50].

The onset of symptoms during childhood or adolescence for many neuropsychiatric disorders suggests a developmental component for their pathogenesis [21, 28, 44]. While disturbed gamma oscillations were shown for prodromal schizophrenia patients, investigations during earlier developmental periods are difficult to perform in humans [40]. Studies in a mouse model of neuropsychiatric disorders revealed reduced oscillatory activity in low-gamma frequency range in the prefrontal cortex during development [23, 57]. This disturbance resulted from a reduced capability of layer 2/3 pyramidal neurons to drive gamma oscillations, matched the frequency bands and the mechanisms of immature gamma oscillations, and correlated with cognitive abilities [10, 23]. Reduced gamma oscillations in this mouse model most likely arise from an excessive pruning of excitatory synapses due to microglial hyperfunction which results in impaired excitation-inhibition balance and decorrelated network activity [22, 23]. Restoring layer 2/3 microcircuits in the prefrontal cortex by inhibition of microglial hyperfunction rescued immature gamma oscillations and cognitive abilities [23].

These results suggest that the coordination of activity in fast oscillatory rhythms, particularly immature gamma oscillations, is impaired early during development in neuropsychiatric disorders, probably before clinically relevant symptoms can be reliably detected. Further research on the specificity of these alterations is required, but the existing studies indicate a potential for cortical gamma oscillations as an early biomarker for neuropsychiatric disorders [31, 50]. Certainly, research on immature gamma oscillations holds great promise to further our understanding of the pathophysiology of neuropsychiatric disorders.

## Gamma oscillations and activity-dependent network refinement

While the initial formation of the cortex is largely controlled by molecular factors, activity-dependent mechanisms gain importance for the refinement of cortical networks [12, 33]. Genetic and activity-dependent mechanisms do not act in isolation, but activity takes a leading role during postnatal development [12]. Neuronal activity influences a wide range of developmental processes, such as dendritic growth, synapse formation and pruning, and the balancing of excitatory and inhibitory inputs [36, 38]. This raises the question whether altered immature gamma oscillations in neuropsychiatric disorders are merely a consequence of a dysfunctional cortical network or whether they actively contribute to the impairment and thereby to disease progression.

Several studies in mice provide new insight on the potential role of early activity for the maturation of cortical networks and cognitive abilities. Inhibition of the mediodorsal thalamus during adolescence leads to long-lasting impairments in prefrontal cortex function, whereas the same manipulation at older age has acute but no long-lasting impact [7, 47]. Along the same lines, a transient manipulation that increases the activity of cortical pyramidal neurons has long-lasting consequences for the development of the prefrontal cortex when it is performed at the beginning, but not at the end of the second postnatal week [9]. The transient manipulation increases the baseline activity and excitability of pyramidal neurons due to an imbalanced excitation-inhibition ratio [9, 45]. This imbalance most likely results from a mismatch of PV^+^ interneuron-mediated inhibition which balances neuronal activity levels and thereby excitatory inputs [9, 58]. Under physiological conditions, this balance is established during the third postnatal week [25, 58]. Notably, transiently increasing activity altered the number and composition of cortical interneurons, indicating that the network lacks the prerequisites to establish a physiological excitation-inhibition ratio after early manipulation [9]. This process might be regulated by an interplay of interneurons and oligodendrocyte precursor cells, which regulate the apoptosis of cortical interneurons in an activity-dependent manner [26].

These network alterations induced by the transient developmental stimulation of layer 2/3 pyramidal neurons result in impaired coordination of cortical activity in gamma oscillations later in life [9, 45]. Interestingly, the impairment of gamma oscillations only becomes prominent several weeks after the manipulation, when the circuit for the generation of gamma oscillations matures, and is most striking when the animals perform a task that requires the prefrontal cortex [9]. Ultimately, these changes result in impaired performance in cognitive tasks at older age, that are associated with cortical gamma oscillations [9, 45]. This is consistent with an impairment of cognitive flexibility and task associated gamma oscillations in adult mice after transient developmental inhibition of prefrontal PV + interneurons [17].

The stimulation of layer 2/3 pyramidal neurons during the second postnatal week did not simply increase activity but also enhanced the coordination of activity in immature gamma oscillations [9]. It is not yet known whether the long-lasting changes in network activity and cognitive behavior are generally due to the transient increase of activity or specifically due to the synchronization of activity in immature gamma oscillations. However, the tight temporal coordination of neuronal activity during gamma oscillations are likely to have a strong influence on plasticity mechanisms during the refinement of neuronal networks [38]. Impaired gamma oscillations are associated particularly with alterations of the excitation-inhibition ratio in the cortex which is established during postnatal development [6, 25]. Whether immature gamma oscillations actively contribute to the formation of the tight matching of inhibitory inputs to excitatory inputs in individual pyramidal neurons remains to be investigated. Further studies are required to narrow down the developmental periods critical for the establishment of excitation-inhibition balance and to elucidate the mechanisms by which synchronization of neuronal activity in gamma oscillations might contribute to it.

In conclusion, these findings show that coordinated activity patterns do not only provide a valuable information about the development of cortical networks, but can also perturb their normal development. Maybe coordinated activity patterns can even be used for targeted interventions to correct deviations from the normal developmental trajectory. Along these lines, a recent study showed that correcting developmental deficits of glutamatergic transmission in layer 2/3 of the cortex rescues electrophysiological deficits and prevents the development of symptoms in a mouse model of Huntington’s disease later in life [13].

## Conclusions and open questions

Immature gamma oscillations emerge in the cerebral cortex during the second postnatal week in mice and increase their frequency until adolescence. Despite having a slower frequency, similarities of the underlying mechanisms and the continuous developmental trajectory suggest that immature gamma oscillations are the precursor of adult gamma oscillations. This provides new insights on the development of cortical networks, particularly on the maturation of fast-spiking interneurons. Further, it mechanistically links findings on impaired gamma oscillations in mouse models of neuropsychiatric disorders during early life and in adulthood which raises several interesting questions:

- How does the developmental trajectory of gamma oscillations change in neuropsychiatric disorders? Knowing the type and the timeline of deviation from physiological development would help to narrow down the set of potential impairments underlying later-emerging symptoms. Further, it might help to identify developmental periods of particular vulnerability.
- Do the deviations of gamma oscillations differ for specific disorders? Considering the developmental trajectory of immature gamma oscillations increases the chances of identifying features that are specific to certain disorders and could therefore be used as early biomarkers to help with diagnosis.
- Are certain developmental windows better suited for intervention than others? The same intervention can result in very different outcomes when it is performed at different time points in a continuously changing system. Comparison of the physiological and pathological development of gamma oscillations could help identify optimal time windows for specific interventions.

There is a continuously improving understanding of cortical gamma oscillations, their development, and how they relate to the structures of the underlying neuronal networks. With the current knowledge it is hard to predict whether gamma oscillations will be of clinical importance as early biomarkers or for therapeutic intervention. Promising results come from a human study, which identified specific developmental changes of auditory-evoked gamma oscillations in individuals at high genetic risk for psychosis [43]. Irrespective of their potential as biomarkers, understanding the development of gamma oscillations will certainly expand our knowledge on the maturation of cortical networks, as well as the mechanisms underlying pathological deviations.
